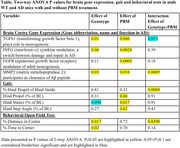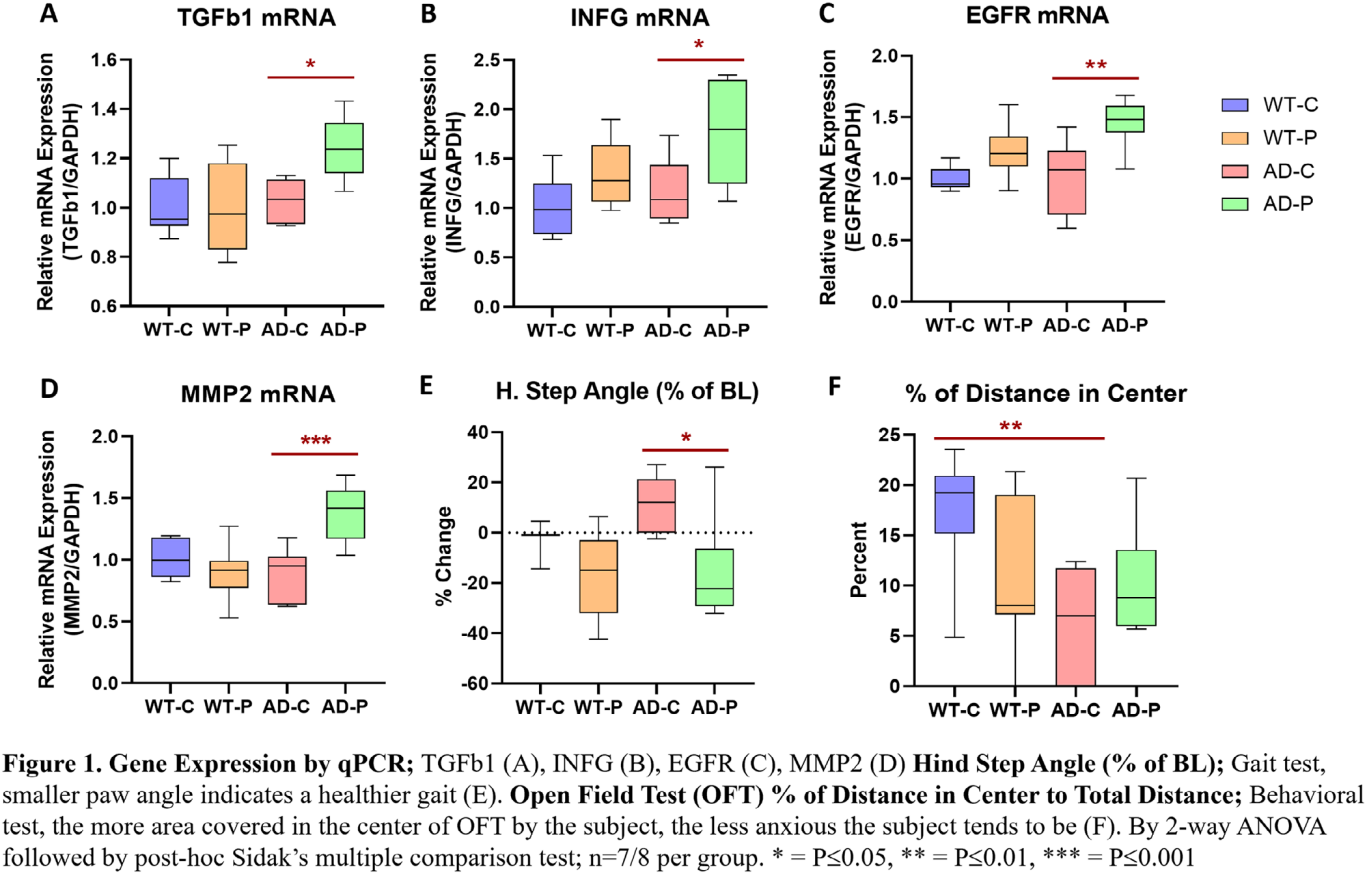# Photobiomodulation Therapy Increases Brain Cortex Neuroprotective Gene Expression and Improves Motor Function in an APP/PS1 Male Mouse Model of Alzheimer’s Disease

**DOI:** 10.1002/alz.087746

**Published:** 2025-01-03

**Authors:** Mackenzie J Barnett, Mahdi Haghkar, Carla Rocha Dos Santos, Ross A McDevitt, Valentina I Zernetkina, Wen Wei, Sunayana Begum Syed, Ondrej Juhasz, Christopher H Morrell, Edward G Lakatta, Olga V Fedorova

**Affiliations:** ^1^ National Institute on Aging/National Institutes of Health (NIA/NIH), Baltimore, MD USA

## Abstract

**Background:**

Photobiomodulation (PBM) therapy, using low intensity near‐infrared light is a noninvasive form of treatment with no side effects can be used to treat Alzheimer’s disease (AD). In a double‐transgenic mouse model of AD (APPswe/PS1dE9), chronic PBM therapy has been shown to reduce Aβ plaques accumulation in specific regions of the brain, including the neocortex and hippocampus. The aim of this study was to analyze the effects of PBM therapy on brain cortex neuroprotective gene expression and behavior in this APPswe/PS1dE9 mouse model.

**Methods:**

Six‐month old male AD (AD‐PBM, n = 7) and wild type litter mate mice (WT‐PBM, n = 8) were exposed to near‐infrared light (wavelength 850 nm, 4.5 J/cm2, 3min/day, 5 days/week) for 6 months. Control mice (AD‐CNT, n = 7; WT‐CNT, n = 7) were placed under the PBM apparatus without turning the light on for 3min/day, 5 days/week. Motor function was assessed with treadmill‐based gait analysis, with metrics including hind propel, hind stance and hind step angle (DigiGait), and open field test (OFT; anxiety‐like behavior) following 6 months of treatment. Gene expression sampled from cortex was analyzed by qPCR. Data were analyzed by 2‐way ANOVA.

**Results:**

In the AD group, PBM therapy significantly increased the expression of neuroprotective genes TGFb1, INFG, EGFR and MMP2, but did not affect the expression of these genes in WT (Fig. 1A‐D). In the gait analysis, PBM groups significantly reduced hind step angle in AD, but not WT (Fig. 1E). In the OFT, genotype had a significant effect on the amount of distance and time spent in the center of the arena (Table); non‐treated WT mice exhibited lower anxiety level vs. non‐treated AD mice (Fig. 1F). PBM did not have a significant effect, but the percent of distance spent in the center out of total distance had an increasing trend (Fig. 1F) indicating a trend in a reduction of anxiety in the AD group. Two‐way ANOVA analysis results are presented in Table 1.

**Conclusion:**

PBM therapy significantly increased neuroprotective cortical gene expression and improved motor function in AD mice. It is possible that PBM treatment contributes to the upregulation of neuroprotective genes that reduced anxiety behavior and improved motor function.